# Swallowing performance of elderly people and sociodemographic, cognitive and language factors

**DOI:** 10.1590/2317-1782/20242022319en

**Published:** 2024-05-31

**Authors:** Jaqueline Cardoso Estácio, Maysa Luchesi Cera, Laura Davison Mangilli

**Affiliations:** 1 Programa de Pós-graduação em Ciências da Reabilitação, Universidade de Brasília – UnB - Brasília (DF), Brasil.; 2 Curso de Fonoaudiologia, Faculdade de Ceilândia, Universidade de Brasília – UnB - Brasília (DF), Brasil.

**Keywords:** Deglutition, Aging, Cognition, Language Tests, Demographic Indicators

## Abstract

**Purpose:**

To analyze the correlation between swallowing, language and cognition performance and describe the sociodemographic data of elderly people without previous neurological disorders.

**Methods:**

Analytical cross-sectional study, with non-probabilistic sample for convenience and data collection by telecall. The aspiration screening test (Yale Swallow Protocol) was used to identify and exclude elderly people at risk of aspiration. Then, sociodemographic data were collected, and instruments were applied: activity of daily living (IADLs), risk of dysphagia (EAT-10), cognitive screening (Mini Mental State Examination - MMSE) and language (Montreal-Toulouse Language Battery - MTL-Brazil).

**Results:**

The sample consisted of 32 elderly people from the Federal District, with a mean age of 69.00±7.73 years and schooling of 10.00±5.60 years. The scores on the EAT-10, MMSE and MTL Battery instruments were altered in four, 22 and 26 elderly, respectively, indicating, in this case, risk of dysphagia, suggestion of cognitive alteration and language alteration. Regarding food, of the total sample, 13 seniors (40%) complained of needing modified food, as well as 10 of these also obtained MMSE scores suggestive of cognitive alteration. When comparing the groups with and without complaints and/or risk of dysphagia, there was no statistically significant difference in relation to sociodemographic, cognitive and language variables. Binary logistic regression models also showed no statistically significant results.

**Conclusion:**

The present study, when correlating the swallowing, language and cognition findings, did not obtain statistically significant results. It was observed that the elderly with swallowing complaints also showed results suggestive of cognitive and language changes in the tests performed, but there was no statistically significant difference in relation to the elderly without complaints or swallowing changes.

## INTRODUCTION

The growth of the older population is a worldwide phenomenon. However, there are fewer resources to ensure healthy aging in Brazil than in developed countries^(1)^. Data from the Brazilian Institute of Geography and Statistics (IBGE) indicate that 10% of the country’s population was 60 years or older in 2018 and estimate that more than a quarter of the population (27%) will be made up of seniors by 2060^(2)^.

Swallowing is one of the functions impaired with aging^(3)^. National and international studies show that 15% of the older population have some type of swallowing difficulty, which can be characterized by changes in any part of the feeding process, such as decreased olfactory and gustatory sensitivity; increased oral transit time; reduced muscle tonus, mobility, and sensitivity of the hyolaryngeal complex; need for consistency adjustments; and prolonged mealtime^(3-5)^.

Older people’s linguistic-cognitive skills are also affected by the aging process, which acts on the cerebral cortex and its functions^(6)^. For instance, their cognitive processing may take longer to perform executive functions, read, comprehension, and memorize^(7)^. They can also have language changes with aging, such as the emergence of paraphasia, increased verbal response latency, and anomia^(7)^. However, the analysis of semantic and lexical aspects of language in older adults shows that their losses or impairments are not always impacted in comparison with adults when controlling for certain sociodemographic factors^(8)^. Age, education, occupation, average family income, and support network have proved to affect the aging process positively or negatively, as they influence cognitive reserve^(9)^.

Furthermore, annual neuropsychological assessments have found that older adults who are in good health and engage in leisure activities regularly were able to maintain their cognitive skills stable until their tenth decade of life. It is worth noting that socioeconomic factors influence older people’s health process more significantly than age itself^(10)^. Therefore, their integration into society and activities of daily living to promote functioning is a protective factor for cognitive functions^(9)^.

It is currently known that aging people can live in harmony with chronic diseases^(11)^. Thus, measures such as health promotion and prevention focusing on the quality of aging should be taken to postpone the onset of complications, prevent clinical worsening and complications, and increase patient and family involvement in the self-care process^(12)^.

The literature has a wide range of publications on older adults’ overall health and aging process, as well as those specifically associated with changes in swallowing, language, and cognition. Most of them focus on older people with neurological impairments, characterizing the functions without analyzing the relationship between these variables.

Individuals with a history of dementia and/or acquired neurological injuries have an impact on swallowing, demonstrating that cognition and language changes can influence swallowing performance^(4)^. Also, sociodemographic variables have been correlated with cognitive and language performance, although swallowing is approached as a characteristic of the sample rather than a focal point of study^(13)^. Nevertheless, characterizing these functions and variables in typical older adults can help plan speech-language-pathologist procedures to promote health and prevent complications in aging.

Since various factors are known to impact health throughout the aging process, there has been a need to correlate swallowing findings in older people with their sociodemographic data such as education, access to healthcare, access to employment, family income, and cognitive and language assessment results.

Hence, this study aimed to analyze the correlation between swallowing, language, and cognitive performance and describe sociodemographic data of older adults without prior neurological changes.

## METHOD

This cross-sectional analytical study was approved by the Human Research Ethics Committee under registration no. CAAE 50832821.8.0000.8093 and evaluation report no. 5.175.892. All participants signed an informed consent form upon agreeing with and consenting to their participation in the research.

The study had a non-probabilistic convenience sample, and it collected data exclusively online via Google Meet video calls, following the guidelines of best practices in speech-language-pathologist teletherapy, as described by the Federal Speech-Language-Hearing Council (Resolution CFFa No. 580/2020). Data were collected from February to September 2022. The sample was recruited through public invitations via promotional flyers and banners, announcements on social media, and online invitations via WhatsApp.

Data were collected remotely due to the ongoing COVID-19 pandemic at the time when this study began, which posed a risk of infection for these older adults when leaving their homes and encountering people who are not part of their usual social circle. Despite the progress of vaccination efforts at the start of the data collection, the research maintained the project as planned during a critical phase of the pandemic, considering the growing trend of remote clinical practices. This justifies the interest in characterizing performance in remote assessments while also prioritizing older people’s safety.

The sample included individuals aged 60 years or older who were interested in participating in the study after a public invitation and understood the informed consent form and task instructions. Almost all participants completed the questionnaires without assistance from a family or professional caregiver. Only one research participant required assistance with using the digital platform, in whose case their caregiver also signed a specific informed consent form.

The study excluded individuals who reported and/or had a previous history of cognitive/neurological impairment complaints or any patient who deemed an aspiration risk in Part I (risk screening for dysphagia) or Part II (brief cognitive screening that might pose a risk for dysphagia) of the Yale Swallow Protocol (YSP) pulmonary aspiration screening test^(14)^. Even though there is no validated Portuguese version of the instrument, the authors of this study are knowledgeable and proficient in English. Hence, they conducted a simple translation of the document and were responsible for its application during data collection, ensuring it was used with integrity. This instrument was chosen according to evidence-based practices, as the guideline for remote assessment of swallowing difficulties recommends using it^(14)^ to screen swallowing in online healthcare during the pandemic.

The YSP is described as a screening tool developed and validated in American English for the risk of pulmonary aspiration, consisting of three stages. In the first one (I), any yes response to one of the following questions would immediately interrupt the test application, namely: inability to remain alert, previous feeding with adapted consistency, use of alternative feeding routes, headrest elevation restriction up to 30º, use of tracheostomy, and oral feeding inability based on medical criteria. Participants who passed the first stage proceeded to the second one (II), which included a brief cognitive screening with time-space orientation and oral inspection, assessing lip sealing at rest, tongue movement range, and facial symmetry (smiling and frowning). They were then asked to sit at 90º and ingest 90 milliliters of water with or without a straw (as they wished) in slow, steady sips in sequence, without pauses, to check for coughing or choking immediately after stopping ingestion. In the third stage (III), the older adult's performance on the YSP was classified as “pass or fail.” Passing the test meant that older people should not have any signs of pulmonary aspiration (such as coughing, throat clearing, or choking) in stage II.

Participants also answered a structured interview to collect sociodemographic data, including age, sex, residence, education level, profession, average family income, health history, and dietary practices to characterize the sample.

After the instruments to include, exclude, and characterize the participants, the protocols described below were applied for the analyses related to the study objectives.

They answered a questionnaire to assess dependence in instrumental activities of daily living through the Lawton and Brody protocol^(15)^, with seven question categories to be classified on a scale from 1 to 3 – the higher the score, the better the participant’s performance in such activities. The results were used to characterize the study sample, and the score indicated dependence when they were lower than two points of the normative cutoff scores.

Dysphagia risk was self-assessed with the Eating Assessment Tool^(16)^ (EAT-10). The score uses a gradual scale from 0 to 4, in which 0 represents “it is not a problem” and 4, “it is a very big problem”, in response to the 10 items in the questionnaire that refer to swallowing complaints. Furthermore, the normative cutoff point, which is three or more, was used to classify the individual's swallowing performance as indicative of dysphagia risk.

The Brazilian version of the Mini-Mental State Examination (MMSE)^(17)^ was applied to screen the participants’ cognitive state. The test has several tasks, including temporal and spatial orientation, immediate memory, attention and calculation, recent memory, naming, repetition, reading, writing, comprehend orders, and copying a drawing. Its score ranges from 0 to 30 points – the lower the score, the worse the cognitive performance. In the copying task, a pentagon was presented via call on the older person's mobile phone screen, with a personalized size for each participant, as they reported being able to see adequately. The score is based on the participant’s education level, according to Brucki et al.^(18)^, following the normative score to define performance with normal or unusual standards, as follows: a minimum of 20 points for illiterates, 25 points for 1 to 4 years of study, 26.5 points for 5 to 8 years, 28 points for 9 to 11 years, and 29 points for 11 or more years^(18)^.

The study also applied the following subtests from the Montreal-Toulouse language assessment battery^(19)^ (MTL-Brazil) to briefly assess language skills: word and sentence repetition, semantic verbal fluency in the “animals” category, phonological/orthographic verbal fluency with the letter M, and oral speech comprehension. The test enables quantitative analysis with points – the higher the score, the better the language performance. The analyses are performed per task, and there is no overall total score in the battery. Each task is scored individually based on a quantitative-qualitative analysis, through the individual calculation of the z-score based on the task score and the normative mean and standard deviation of the Brazilian population according to their education level. The study followed the normative score to define unusual results.

Choosing specific linguistic tests that cover the different linguistic levels (phonological, morphosyntactic, and semantic) is essential to obtain a complete and detailed view of one's linguistic skills. The research used these language tests because it focused on linguistic-cognitive screening, in which case the literature demonstrates that the greatest sensitivity for identifying subtle changes in older people occurs when performing tasks such as verbal fluency and oral comprehension^(20)^.

The average application time for all tests was 1 hour, with a minimum time of 50 minutes and a maximum of 1 hour and 40 minutes. All participants used their mobile phones to make the call.

### Proposed statistical analysis

The data were analyzed descriptively and inferentially with SPSS 25.0 software. The significance level was set at 5% for inferential analyses.

Measures of central tendency (mean and median), variability (standard deviation), and position (minimum, maximum, and quartiles one and three) were calculated in the descriptive analysis of quantitative variables. The absolute frequency and relative percentage frequency were calculated in the descriptive analysis of the qualitative variables.

The inferential analysis of the comparison of non-normal quantitative and ordinal qualitative variables between two independent groups was performed with the Mann-Whitney Test.

The correlation between non-normal quantitative and ordinal qualitative variables was performed with the Spearman correlation test. The inferential association analysis between nominal qualitative variables was performed with the chi-square test.

A multiple linear regression model was also used to predict the quantitative dependent variable. The stepwise method was used for selecting the independent variables. A binary logistic regression model was developed to predict the binary nominal qualitative dependent variable. The forward selection method (likelihood ratio) was used.

## RESULTS

The study sample had a total of 32 older adults, 25 of whom were females, with a mean age of 69.00±7.73 years. Only four participants were 80 years or older. The mean education level in years was 10.00±5.60, and the mean family income was R$ 2,646±1,001.

As shown in Table 1, all participants were retired, lived in the Federal District, and had some chronic disease. Most of them (n = 23) were independent in activities of daily living.

**Table 1:** Descriptive variables of the sample (n = 32)

Sex	n	%
Females	25	78.2
Males	7	21.8
Previous occupation		
Manual work	16	50.0
Intellectual work (administrative, legal, and/or academic)	9	28.2
Stay-at-home wives	7	21.8
Mean family income, in reais		
Above 3,300 reais	22	68.7
From 1,100 to 2,200 reais	9	28.1
From 2,200 to 3,300 reais	1	3.2
Place of residence		
Other	7	22.0
Ceilândia	6	18.9
Lago Norte	3	9.5
Gama	2	6.2
Cruzeiro Novo	2	6.2
Asa Norte	2	6.2
Águas Claras	2	6.2
Vicente Pires	2	6.2
Samambaia	2	6.2
Brazlândia	2	6.2
Santa Maria	2	6.2
Health history^*^		
Metabolic disease	19	59.3
Cardiovascular disease	14	43.7
COVID-19	11	34.3
Oncological disease	6	18.8
Respiratory disease	4	12.5
Gastrointestinal disease	4	12.5
Endocrine disease	3	9.3
Rheumatologic disease	2	6.2
Activities of daily living		
Independence	23	71.8
Partial dependence	9	28.1
Total dependence	0	0

* Participants could report more than one disease

Regarding diet, 28 participants prepared their meals, and half of the sample took more than 30 minutes to eat, with a mean of 3.81±0.47 meals a day.

The findings of the dietary complaints are described in Table 2, which shows that 13 older adults needed a modified daily diet, with soft and moist solids. Only four individuals had unusual scores (3 or more points) in the self-reported swallowing difficulties test (EAT-10).

**Table 2:** Self-reported eating complaints (n = 32)

	n	%
Complaint of dysphagia for thin liquids	3	9
Complaint of dysphagia for solid foods	2	6
Feed on modified foods (soft moist solid foods)	13	40
Complaint of difficulty in ingesting medications	5	15
Complaint of difficulty in masticating solid foods	3	9
Unusual EAT-10 results (3 or more points)	4	12

Caption: EAT-10 = Eating Assessment Tool

The mean cognition score in the MMSE was 26.22±2.49 points, as seen in Table 3. Most individuals (n = 22) had scores below expectations, according to the cutoff for their education levels. Furthermore, 10 out of the 13 participants with swallowing complaints requiring modified food also had lower MMSE scores for their education level.

**Table 3:** Spearman correlation test between EAT-10 results and demographic, cognitive, and linguistic variables (n = 32)

	EAT-10			
	**M**	**SD**	**ρ**	**p-value**
Age	69.38	7.73	-0.125	0.496
Education level (in years)	10.41	5.60	-0.103	0.575
MMSE	26.22	2.49	-0.249	0.170
Word repetition^*^	-2.52	3.15	-0.072	0.694
Semantic verbal fluency*	-0.41	0.79	-0.262	0.148
Phonological verbal fluency*	-0.84	1.23	-0.137	0.453
Oral speech comprehension*	0.11	0.87	-0.037	0.842

* z-score according to education level

Caption: M = mean; SD = standard deviation; EAT-10 = Eating Assessment Tool; MMSE = Mini Mental State Examination

In the MTL-Brazil language battery tasks, such as word repetition, phonological and semantic verbal fluency, and oral speech comprehension, only in the latter did all participants have values within normal limits according to their education level. As for the other tasks, 26 participants had lower scores.

Moreover, only one of the 13 participants with eating complaints did not have abnormal results in the language tests. The other 12 had a z-score indicating changes in at least one language task.

As previously pointed out, four participants had unusual EAT-10 results. When comparing questions related to food with those related to cognition and language, all participants with unusual EAT-10 results also had lower MMSE scores and at least one language task whose z-score indicated changes.

However, no statistically significant correlation was found between EAT-10 results and age, years of education, cognitive screening test results, and language task results.

The non-parametric Mann-Whitney test assessed older people with and without unusual EAT-10 results and found no statistically significant difference between these two groups regarding age, education level, MMSE results, and MTL-Brazil battery task results, such as word repetition, semantic verbal fluency, phonological verbal fluency, and oral speech comprehension, according to the p-values shown in Table 4.

**Table 4:** Mann-Whitney test for the group with unusual EAT-10 results and complaints of needing modified foods with the other study variables (n = 32)

	Need for modified foods				EAT-10			
	**M**	**SD**	**U**	**p-value**	**M**	**SD**	**U**	**p-value**
Age	68.5	8.7	99.0	0.346	65.5	3.5	38.5	0.317
Education level (in years)	9.1	5.0	96.5	0.296	12.5	3.7	41.0	0.389
MMSE	25.4	2.4	75.0	0.060	26.5	1.7	53.0	0.863
Word repetition^*^	-2.3	3.1	118.0	0.832	-1.2	1.5	43.5	0.474
Semantic verbal fluency*	-0.6	0.7	92.5	0.234	-0.4	0.6	55.0	0.954
Phonological verbal fluency*	-1.2	1.0	91.0	0.212	-0.8	1.1	56.0	1.000
Oral speech comprehension*	0.0	0.8	95.5	0.278	0.3	0.5	49.0	0.687

* z-score according to education level

Caption: M = mean; SD = standard deviation; U = value of the comparison statistic using the Mann Whitney test; EAT-10 = Eating Assessment Tool; MMSE = Mini Mental State Examination

Furthermore, there was also no statistically significant difference between the complaint of the need for modified food with the age, education level, MMSE, word repetition, semantic verbal fluency, phonological verbal fluency, and oral speech comprehension tasks, according to demonstrated p-values.

Hence, it cannot be inferred that the presence of swallowing complaints is correlated with age and the above test results in older people with and without such complaints.

Binary logistic regression models were developed to predict dependent variables such as signs of dysphagia for liquids and solids, need for modified food, difficulty taking medicine, difficulty masticating, EAT-10 (considering the cutoff for unusual results), and YSP, based on independent variables such as age, sex, education level, MMSE, and MTL-Brazil battery tasks. A multiple linear regression model was also developed to predict the dependent variable EAT-10 based on independent variables such as age, sex, education level, MMSE, and MTL-Brazil battery. No model obtained statistically significant results.

## DISCUSSION

This study aimed to describe swallowing findings, sociodemographic variables, and cognitive and language performance and verify the association between these factors. It was observed that almost half of the sample had eating complaints, 68% had lower MMSE scores, and 81% had unusual results in at least one language task. Despite the absence of statistically significant associations that prove the correlation between variables – which will be discussed below –, it was found that the same older people who complained of eating difficulties also had scores suggestive of cognitive and linguistic changes.

The 32 participants reported having an average of just four meals a day, compatible with a study that found the same average in Southern Brazil, considering this amount insufficient to meet human nutritional needs^(21)^. As for eating complaints, almost half of the sample (40%) reported the need for modified foods and complained of difficulty masticating and swallowing solids, liquids, and medications. Some previously described factors may be related to food, such as decreased appetite with advancing age, decreased sense of smell and taste, medication effects on taste and appetite, difficulties preparing meals for others, and discouragement in preparing their meals because they live alone, besides sociodemographic factors such as having money to buy food^(21,22)^.

Furthermore, the eating difficulties reported in the present study can be justified by age, a demographic variable that is a risk factor for the development of dietary adjustments^(4)^. Studies show that around 13% of people over 65 years old have swallowing difficulties, and this number can rise to 50% when considering those with cognitive impairments^(22)^. Such swallowing difficulties, characterized as presbyphagia, change the process without necessarily causing laryngotracheal penetration/aspiration, despite increasing the risk of occurrence. However, these difficulties can impact nutritional aspects^(23)^.

Among the complaints reported by the older people in this study, there was a greater frequency (40%) of needing modified food consistency to soft and moist solids. This need may be related to factors observed in older adults, such as decreased tongue strength, increased mastication time, decreased oropharyngeal pressure for bolus ejection, greater presence of residues in the oral cavity and pharynx, greater occurrence of spontaneous-clearing laryngeal penetration, and decreased esophageal motility^(24)^.

Moreover, a recent study assessed older people’s swallowing through videofluoroscopy and observed that almost half of the participants over 85 years old had dysphagia, even without previous underlying diseases, and that presbyphagia was justified by age-related sarcopenia^(25)^. It also found that the older the person, the more swallowing changes they had, with a cumulative effect^(25)^. Only four older adults in the present study had an unusual EAT-10 self-assessed score for the presence of dysphagia risk. The lower proportion of older people with signs suggestive of dysphagia in this study can be justified by their age, which will be discussed below.

Even though the present study had few participants with changes in EAT-10, a study that applied this protocol to typical older adults found a positive correlation between age and the EAT-10 score, demonstrating its relevance for identifying swallowing difficulties^(26)^. Again, the present study results had no statistically significant correlation between EAT-10 scores and age. This difference from the literature may be due to the age of the sample, predominantly made up of younger older people, while only three of them were over 85 years old. Thus, the results should be mainly considered for older adults aged up to 85 years, given the small sample and its few older people at a more advanced age.

Furthermore, the lower proportion of older adults at risk of dysphagia may be related to the participants’ mean education level^(27)^ (10 years) and income (above the average for the general population)^(28)^ and the predominance of women in the sample, as these variables are associated with better health status^(9,10)^.

It should be added that almost all individuals with eating complaints had an MMSE score suggestive of changes, according to Brazilian normative data^(18)^, and lower scores in at least one MTL battery language task. Cognition is known to be part of the swallowing control process (mainly during the oral phase of swallowing), food recognition, and sensations such as eating pleasure and satiety; hence, individuals with diagnosed cognitive changes (e.g., dementia) may have impaired food security and nutrition^(27)^. However, expected swallowing changes in typical older people are more related to muscular aging than cognitive control^(24)^. The study did not identify a statistically significant correlation between the eating and swallowing condition and the cognitive and language status. Thus, it is believed that the eating complaints and the EAT-10 scores suggestive of swallowing changes in this study are mainly related to muscle condition since the participants whose MMSE scores were suggestive of cognitive decline had scores quite near the normative data.

Recent studies on presbyphagia have focused on the physiological/anatomical changes associated with aging. However, the exclusive focus on these transformations without properly considering the influence of cognitive skills in this process has been increasingly questioned^(24-27)^. Although structural and functional changes in the digestive system, from the mouth to the stomach, play a significant role in presbyphagia, neglecting the interaction between cognitive aspects and swallowing may limit a comprehensive understanding of the challenges faced by older adults.

However, studies demonstrate a correlation between cognition and swallowing – despite the literature relating muscle aging with swallowing findings and despite the present research not finding statistically significant data when correlating the variables. A study carried out by Maniaci and collaborators^(29)^ demonstrated that 44% of the group with lower MMSE scores had dysphagia, with statistically significant data when compared to the control group. Furthermore, control subjects with better MMSE scores (despite being already within expected normal values for their education level and age) performed better in swallowing^(29)^.

Specifically, regarding cognition, 22 participants had MMSE scores below the expected for their education level^(18)^. Other studies with neurotypical older adults also found MMSE scores suggestive of cognitive changes^(30,31)^. The present research found 68% of older people with a score suggestive of cognitive changes, while the cited studies identified respectively 55% and 16% of older people with this same clinical condition. On the other hand, a study with older people from the same region as this study also identified a high prevalence of older people (80%) with a clinical condition suggestive of cognitive changes, likewise through the MMSE^(32)^. Thus, it is believed that sociodemographic characteristics can justify cognitive results. Nonetheless, the instrument's normative data were obtained with a sample from another region of the country, which can further highlight the relevance of demographic variables in the analysis of cognitive clinical conditions. Furthermore, applying the tests online can cause comprehension difficulties subtle enough to lead to unusual results – which is a limitation of this study.

Moreover, studies indicate that MMSE results suggestive of changes do not necessarily mean that these older adults will develop dementia since the development of these changes also depends on interference in activities of daily living, family life, and mood^(30)^.

Furthermore, the older people’s mean education level in the present sample was 10.00±5.60 years, and their mean income was better than that of Brazilians in general. This could explain a better linguistic-cognitive performance, based on the theory of cognitive reserve, and positively interfere with swallowing performance – even being a compensating factor for possible eating difficulties, as cognitive reserve delays cognitive decline symptoms related to the disease^(33)^.

It must be highlighted that carrying out this research online may have influenced the responses since the chosen protocols were initially described for in-person application. Moreover, virtual environments may hinder the test performance of older people with medium and low education levels due to their unfamiliarity with technology – even though this variable was controlled, as they could be assisted by a companion, and the participants’ results were compatible with the previous literature.

Furthermore, since all protocols used in this study are screening tests, more robust and detailed data on cognition and its specific aspects (such as executive functions, working memory, types of attention, and so forth) and objective swallowing examinations must be taken into consideration, as changes in any of these skills could subtly interfere with the cognitive processing of swallowing, as demonstrated in the literature^(29)^.

It is also suggested that future studies, especially in virtual mode, include larger samples.

## CONCLUSION

This study found no statistically significant correlation between swallowing, language, and cognition. Nevertheless, it was observed that older adults with swallowing complaints also had test results suggestive of cognitive and language changes. 
