# Supplementary material for: Swallowing performance of elderly people and sociodemographic, cognitive and language factors
Source: Codas. 2024 May 31;36(4):e20220319. doi: 10.1590/2317-1782/20242022319en (PMC11189153; doi:10.1590/2317-1782/20242022319en)
Supplement: Translation

# Translation: Desempenho de deglutição de idosos e seus fatores sociodemográficos, cognitivos e de linguagem

**DOI:** 10.1590/2317-1782/20242022319pt

**Authors:** Jaqueline Cardoso Estácio, Maysa Luchesi Cera, Laura Davison Mangilli

**Affiliations:** 1 Programa de Pós-graduação em Ciências da Reabilitação, Universidade de Brasília – UnB - Brasília (DF), Brasil.; 2 Curso de Fonoaudiologia, Faculdade de Ceilândia, Universidade de Brasília – UnB - Brasília (DF), Brasil.

**Keywords:** Deglutição, Envelhecimento, Cognição, Testes de Linguagem, Indicadores Demográficos

## Abstract

**Objetivo:**

Analisar a correlação entre o desempenho de deglutição, linguagem e cognição e descrever os dados sociodemográficos de idosos sem alterações neurológicas prévias.

**Método:**

Estudo transversal analítico, com amostra não-probabilística por conveniência e coleta de dados por telechamada. Foi aplicado o teste de triagem de broncoaspiração (Yale Swallow Protocol) para identificação e exclusão dos idosos com risco de broncoaspiração. Em seguida, realizou-se coleta de dados sociodemográficos e aplicação dos instrumentos de: atividade de vida diária (AIVDs), risco de disfagia (EAT-10), rastreio cognitivo (Mini Exame do Estado Mental – MEEM) e linguagem (Bateria Montreal-Toulouse de Linguagem – MTL-Brasil).

**Resultados:**

A amostra foi composta por 32 idosos do Distrito Federal, com média de idade de 69,00±7,73 anos e de escolaridade de 10,00±5,60 anos. Os escores nos instrumentos EAT-10, MEEM e Bateria MTL apresentaram-se alterados em quatro, 22 e 26 idosos, respectivamente, indicando, nesse caso, risco de disfagia, sugestão de alteração cognitiva e alteração da linguagem. Sobre a alimentação, do total da amostra, 13 idosos (40%) apresentaram queixa de necessidade de comida modificada, bem como 10 desses também obtiveram escore no MEEM sugestivo de alteração cognitiva. Ao comparar os grupos com e sem queixa e/ou risco de disfagia, não houve diferença estatisticamente significante em relação às variáveis sociodemográficas, cognitivas e de linguagem. Os modelos de regressão logística binária também evidenciaram resultados sem significância estatística.

**Conclusão:**

O presente estudo, ao correlacionar os achados de deglutição, linguagem e cognição, não obteve resultados estatisticamente significantes. Observou-se que os idosos com queixa de deglutição também apresentaram resultados sugestivos de alteração cognitiva e de linguagem nos testes realizados, mas não houve diferença estatisticamente significante em relação aos idosos sem queixa ou alteração de deglutição.

## INTRODUÇÃO

O crescimento da população idosa é um fenômeno mundial. No Brasil há menos subsídios que favoreçam o envelhecimento saudável quando comparado a países desenvolvidos^(1)^. A porcentagem de pessoas com 60 anos ou mais no Brasil em 2018 era de 10% da população, segundo os dados do Instituto Brasileiro de Geografia e Estatística (IBGE) e a projeção é que, para 2060, mais de ¼ da população seja composta por idosos, com porcentagem de 27%^(2)^.

Uma das funções que sofre prejuízo com o decorrer do envelhecimento é a deglutição^(3)^. Estudos internacionais e nacionais mostram que 15% da população idosa apresenta algum tipo de dificuldade de deglutição, que pode ser caracterizada por alterações em qualquer parte do processo de alimentação, como diminuição da sensibilidade olfativa e gustativa, tempo de trânsito oral aumentado, redução de tônus, mobilidade e sensibilidade do complexo hiolaríngeo, necessidade de ajustes de consistências e tempo de refeição prolongado^(3-5)^.

As habilidades linguístico-cognitivas do idoso também sofrem impactos do processo de envelhecimento, que age sobre o córtex cerebral e suas funções^(6)^. Os idosos podem apresentar tempos maiores de processamento cognitivo para desempenhar funções executivas, bem como ler, compreender e memorizar, por exemplo^(7)^. Durante o envelhecimento, pode haver mudanças também na linguagem, com surgimento de parafasias, aumento na latência de resposta verbal e anomias^(7)^. No entanto, ao analisar aspectos semânticos e lexicais da linguagem do idoso, é possível observar que nem sempre há perdas ou prejuízos expressivos ao comparar com sujeitos adultos, quando controlados alguns fatores sociodemográficos^(8)^. Observa-se que idade, escolaridade, profissão, renda média familiar e rede de apoio geram impactos positivos ou negativos no processo de envelhecimento, uma vez que influenciam na reserva cognitiva^(9)^.

Além disso, constatou-se que idosos que se encontram em boas condições de saúde e possuem atividades de lazer na rotina, ao passarem por uma avaliação neuropsicológica anual, conseguiram manter suas habilidades cognitivas estáveis até a décima década de vida. Vale ressaltar que os fatores socioeconômicos demonstram exercer uma influência mais significativa no processo de saúde dos idosos do que a própria idade^(10)^. Sendo assim, a inserção do idoso na sociedade e em atividades de vida diária, que visam e promovem a funcionalidade, demonstra ser fator de proteção das funções cognitivas^(9)^.

Atualmente, já se sabe que é possível conviver em harmonia com doenças crônicas no envelhecimento^(11)^. Dessa forma, medidas com enfoque na qualidade do envelhecimento da pessoa idosa, como promoção e prevenção, devem ser adotadas, visando adiar o surgimento de complicações, prevenir exacerbações e complicações clínicas, além de aumentar o envolvimento do paciente e da família no processo de autocuidado^(12)^.

Observa-se, ainda, na literatura, vasta amplitude de publicações acerca da saúde geral do idoso e do processo de envelhecimento e, aqueles que estão associados especificamente às alterações de deglutição, de linguagem e de cognição. Trata-se em sua grande maioria de idosos com comprometimentos neurológicos, com caracterização das funções, sem análise da relação entre essas variáveis.

Observa-se impacto na deglutição em casos de pessoas com história de demências e/ou lesões neurológicas adquiridas, demonstrando que, a cognição e a linguagem, quando alteradas, podem influenciar no desempenho de deglutição^(4)^. Já quando se trata das variáveis sociodemográficas, observa-se a correlação delas com desempenho cognitivo e de linguagem, e a deglutição surge como caracterização da amostra e não como ponto de estudo^(13)^. Porém, a caracterização destas funções e variáveis em idosos típicos pode contribuir para o planejamento de ações fonoaudiológicas no processo de promoção e prevenção de complicações no envelhecimento.

Uma vez que já é de conhecimento que a saúde ao longo do processo de envelhecimento pode sofrer impactos de diversos fatores, viu-se a necessidade de correlacionar, em idosos, os achados de deglutição com as informações sociodemográficas como escolaridade, acesso à saúde, acesso à emprego, renda familiar; e com os dados obtidos a partir da avaliação cognitiva e de linguagem.

Dessa forma, o objetivo deste estudo foi analisar a correlação entre o desempenho de deglutição, linguagem e cognição e descrever os dados sociodemográficos de idosos sem alterações neurológicas prévias.

## MÉTODO

Estudo do tipo analítico transversal, aprovado pelo Comitê de Ética e Pesquisa em Seres Humanos sob o registro nº CAAE 50832821.8.0000.8093 e parecer nº 5.175.892. Todos os participantes assinaram o Termo de Consentimento Livre e Esclarecido (TCLE), estando de acordo e consentindo a sua participação na pesquisa.

Trata-se de amostra não-probabilística por conveniência, realizada exclusivamente por meio de coleta de dados *online* via telechamada via *Google Meet,* de acordo com as diretrizes de boas práticas em telefonoaudiologia, descrito pelo Conselho Federal de Fonoaudiologia (Resolução CFFa nº 580/2020). A presente coleta ocorreu no período de fevereiro a setembro de 2022. A forma de captação da amostra de coleta deu-se por meio de convite público via folders de divulgação e banners, exposição via redes sociais e convites *online* via Whatsapp.

A escolha pela coleta remota se deu devido à ainda presente pandemia de COVID-19 na época do início deste estudo, bem como o risco de infecção destes idosos ao saírem de casa e o contato com pessoas que não são de convívio social comum. Apesar do avanço da vacinação, na época do início da coleta do estudo, a presente pesquisa manteve o projeto planejado em um período crítico da pandemia, devido ao crescimento da clínica remota que justifica o interesse pela caracterização do desempenho em avaliações nesse formato, além de considerar a segurança do idoso.

Foram incluídos na amostra indivíduos de 60 anos ou mais, que referiram interesse em participar do estudo após convite público, e que demonstraram compreender o TCLE, bem como as instruções das tarefas propostas. Quase todos os idosos responderam os questionários sem ajuda de um cuidador familiar ou profissional – apenas um participante da pesquisa necessitou de auxílio para uso da plataforma digital, e, nesse caso, seu acompanhante também assinou TCLE específico.

Além disso, foram excluídos da pesquisa aqueles que apresentaram relato e/ou histórico de comprometimento cognitivo/neurológico prévio como queixa referida e também aqueles que apresentaram qualquer alteração na parte I (rastreio de risco de disfagia) ou II (breve rastreio cognitivo que poderia impactar em risco de disfagia) do teste de rastreio de broncoaspiração *Yale Swallow Protocol* ^(14)^ (YSP). Apesar de não existir a versão validada do instrumento para a língua portuguesa, as autoras do estudo, que possuem conhecimento e aptidão para o uso da língua-inglesa realizaram a tradução simples do documento e foram as responsáveis pela sua aplicação no momento da coleta de dados, garantindo a integridade de seu uso. A escolha por este instrumento seguiu a prática baseada em evidência, sendo este o instrumento que consta no *guideline* para atendimento virtual de dificuldades de deglutição^(14)^, no qual o protocolo é realizado como forma de triagem da deglutição diante do contexto de pandemia no atendimento virtual.

O YSP é descrito como uma triagem para risco de broncoaspiração, produzida e validada no inglês-americano. A triagem por meio do YSP foi composta por três etapas. Na primeira etapa (I), qualquer resposta sim para uma das questões a seguir interromperia imediatamente a aplicação do teste, sendo elas: incapacidade de permanecer alerta, alimentação prévia com consistência adaptada, uso de vias alternativas de alimentação, restrição de cabeceira até 30º, uso de traqueostomia e impossibilidade de via oral por critério médico. O participante que passou na primeira etapa, seguiu para a segunda etapa (II), que inclui a realização de rastreio cognitivo breve com orientação tempo espacial e inspeção oral com avaliação do selamento labial em repouso, amplitude dos movimentos de língua e simetria facial (sorriso e face franzida). Após, foi solicitado que o participante, sentado à 90º, ingerisse um copo de água de 90 mililitros com canudo ou não (por escolha do indivíduo) em gole livre (sequenciais) de maneira lenta e constante, porém, sem pausas, a fim de verificar a presença de tosse ou engasgo imediatamente após parar a ingestão. Na terceira etapa (III), o desempenho do idoso no YSP foi classificado como “passa ou falha”. Para considerar o idoso que passou no teste, este deveria não apresentar nenhum sinal de broncoaspiração como tosse, pigarro ou engasgo ao realizar o passo II do protocolo.

Além disso, os participantes passaram por entrevista dirigida a fim de coletar os dados sociodemográficos, que contemplam dados como idade, gênero, local de residência, escolaridade, profissão, renda média familiar, histórico de saúde e práticas alimentares para caracterização da amostra.

Após a aplicação dos instrumentos para inclusão e exclusão e caracterização dos participantes, realizou-se a aplicação dos protocolos que serão descritos a seguir, usados para as análises referentes aos objetivos deste estudo.

Foi aplicado o questionário para avaliação da dependência nas Atividades Instrumentais de Vida Diária (AIVDs) por meio da aplicação do protocolo de Lawton e Brody^(15)^, composto por 7 categorias de perguntas, que deveriam ser classificadas em uma escala de um (1) a três (3), na qual quanto maior a pontuação obtida, melhor o desempenho do participante na realização das AIVDs. Os resultados obtidos foram utilizados para caracterização da amostra de estudo e a pontuação obtida indicou dependência quando menor que dois pontos das notas normativas de corte.

A autoavaliação do risco de disfagia foi realizada por meio do protocolo *Eating Assessment Tool* ^(16)^ (EAT-10). A pontuação apresenta uma escala gradual de zero a quatro, na qual zero (0) representa “não é um problema” e quatro (4) “é um problema muito grande”, em resposta às dez perguntas presentes no questionário que se referem a questões de queixas de deglutição. Além disso, foi utilizado o ponto de corte normativo para classificar o desempenho de deglutição do indivíduo como indicativo de risco de disfagia, que é de três ou mais.

No que se refere ao estado cognitivo dos participantes, foi aplicado o Mini Exame do Estado Mental (MEEM) versão brasileira^(17)^, para triagem. O teste é composto por diversas tarefas, dentre elas a orientação temporal e espacial, memória imediata, atenção e cálculo, memória recente, nomeação, repetição, leitura, escrita, compreensão de ordem e cópia de desenho. Pontua-se de 0 a 30 pontos, sendo que, quanto menor a pontuação, pior o desempenho cognitivo apresentado. Na etapa de cópia do desenho, a imagem do pentágono foi apresentada via chamada na tela do celular do idoso, de tamanho personalizado para cada participante (em que ele referisse conseguir enxergar adequadamente). Sendo assim, a pontuação obtida é relacionada à escolaridade, conforme Brucki et al.^(18)^; nesse caso, respeitou-se a pontuação normativa para definição do desempenho com alteração ou dentro do padrão de normalidade, sendo minimamente 20 pontos para analfabetos, 25 pontos para 1 a 4 anos de estudo, 26,5 pontos para 5 a 8 anos, 28 pontos para 9 a 11 pontos e 29 pontos para 11 anos ou mais^(18)^.

Ademais, realizou-se a aplicação de subtestes da bateria Montreal-Toulouse de avaliação da linguagem^(19)^ (MTL-Brasil), visando a avaliação breve das habilidades linguísticas. Foram aplicadas as tarefas de repetição de palavras e frases; fluência verbal semântica na categoria animais e fluência verbal fonológica/ortográfica com a letra M e compreensão oral do texto. O teste possibilita a análise quantitativa por sistema de pontos, no qual quanto maior a pontuação, melhor o desempenho de linguagem. As análises são feitas por tarefa e não há pontuação total geral obtida na bateria, uma vez que cada tarefa é pontuada individualmente a partir de análise quanti-qualitativa, por meio do cálculo individual do z-escore baseado no escore obtido e na média normativa e desvio padrão da população brasileira conforme sua escolaridade; além disso, respeitou-se a pontuação normativa para definição da presença de alteração.

A escolha de testes linguísticos específicos que abrangem os diferentes níveis linguísticos (fonológico, morfossintático e semântico) é fundamental para obter uma visão completa e detalhada das habilidades linguísticas de um indivíduo. A escolha dos testes de linguagem descritos, nesta pesquisa, deve-se ao fato de que, devido ao enfoque se tratar do rastreio linguístico-cognitivo, a literatura demonstra que nestes casos, a maior sensibilidade de identificação de alterações sutis em idosos ocorre na execução de tarefas como fluência verbal e a compreensão oral^(20)^.

O tempo médio de aplicação de todos os testes foi de uma hora, com tempo mínimo de 50 minutos e máximo de 1 hora e 40 minutos. Todos os participantes utilizaram o aparelho de celular para a realização da tele chamada.

### Análise estatística proposta

Os dados foram analisados de forma descritiva e inferencial utilizando-se o software SPSS 25.0. Foi considerado um nível de significância de 5% para as análises inferenciais.

Na análise descritiva das variáveis quantitativas foram calculadas as medidas de tendência central (média e mediana), variabilidade (desvio-padrão) e posição (mínimo, máximo, primeiro e terceiro quartis). Na análise descritiva das variáveis qualitativas foram calculadas a frequência absoluta e a frequência relativa percentual.

A análise inferencial de comparação das variáveis quantitativas não-normais e qualitativas ordinais entre dois grupos independentes foi realizada com o Teste de Mann-Whitney.

A correlação entre as variáveis quantitativas não-normais e qualitativas ordinais foi realizada com o Teste de Correlação de Spearman. Quanto à análise inferencial de associação entre as variáveis qualitativas nominais, essa foi realizada com o teste Qui-quadrado.

Utilizou-se, ainda, modelo de regressão linear múltipla para previsão da variável dependente quantitativa. O método de seleção das variáveis independentes foi o *stepwise*. Foi elaborado um modelo de regressão logística binária para previsão da variável dependente qualitativa nominal binária. Utilizou-se o método de seleção *forward* (razão de verossimilhança).

## RESULTADOS

A amostra deste estudo foi composta por um total de 32 idosos, sendo 25 do gênero feminino, com média de idade de 69,00±7,73 anos. É importante destacar que somente quatro participantes possuíam 80 anos de idade ou mais. A média de escolaridade, em anos, foi de 10,00±5,60, com média de renda média familiar de R$ 2.646±1.001 reais.

Conforme demonstra a Tabela 1, todos os participantes eram aposentados e residiam no Distrito Federal (DF), apresentavam alguma doença crônica e na sua maioria (n=23) possuíam independência para as atividades de vida diária.

**Tabela 1:** Variáveis descritivas da amostra (n = 32)

Gênero	n	%
F	25	78,2
M	7	21,8
Atividade laboral prévia		
Caráter físico/braçal	16	50
Caráter intelectual (administrativo, jurídico e/ou acadêmico)	9	28,2
“Do lar”	7	21,8
Renda média familiar em reais		
Acima de 3300 reais	22	68,7
Entre 1100 e até 2200 reais	9	28,1
Acima de 2200 e até 3300 reais	1	3,2
Local de residência		
Outros	7	22
Ceilândia	6	18,9
Lago Norte	3	9,5
Gama	2	6,2
Cruzeiro Novo	2	6,2
Asa Norte	2	6,2
Águas Claras	2	6,2
Vicente Pires	2	6,2
Samambaia	2	6,2
Brazlândia	2	6,2
Santa Maria	2	6,2
Histórico de saúde^*^		
Doença metabólica	19	59,3
Doença cardiovascular	14	43,7
Covid-19	11	34,3
Doença oncológica	6	18,8
Doença respiratória	4	12,5
Doença gastrointestinal	4	12,5
Doença endocrinológica	3	9,3
Doença reumatológica	2	6,2
Atividades de vida diária		
Independência	23	71,8
Dependência parcial	9	28,1
Dependência total	0	0

* Os participantes poderiam referir mais de uma doença

Legenda: F = feminino; M = masculino

No que se refere à alimentação, 28 participantes preparavam a própria refeição e metade da amostra levava mais que 30 minutos para se alimentar, com uma média de 3,81±0,47 refeições ao dia.

Além disso, estão descritos na Tabela 2 os achados das queixas alimentares apresentadas, na qual é possível observar que 13 idosos necessitavam de alimentação modificada, com sólidos macios e úmidos, no dia a dia. Já no que se refere ao teste autorreferido de dificuldades de deglutição (EAT-10), apenas quatros indivíduos apresentaram escore alterado (três pontos ou mais).

**Tabela 2:** Queixas alimentares autorreferidas (n = 32)

	n	%
Queixa de disfagia para líquidos ralos	3	9
Queixa de disfagia para sólidos	2	6
Alimentam-se com comida modificada (sólidos úmidos e macios)	13	40
Queixa de dificuldade para ingerir medicamentos	5	15
Queixa de dificuldade de mastigação de sólidos	3	9
Resultado EAT-10 alterado (3 pontos ou mais)	4	12

Legenda: EAT-10 = Eating Assessment Tool

No que tange à cognição, conforme observado na Tabela 3, a média de pontuação obtida no MEEM foi de 26,22±2,49 pontos. Dessa pontuação, a maioria dos indivíduos (n=22) apresentaram valores abaixo do esperado para a nota de ponto de corte da sua escolaridade. Além disso, dos 13 participantes que apresentaram queixa de deglutição com necessidade de comida modificada, dez deles também apresentaram escore do MEEM alterado para a escolaridade.

**Tabela 3:** Teste de correlação de Spearman entre resultados do EAT-10 e as variáveis demográficas, cognitivas e linguísticas (n = 32)

	EAT-10			
	**M**	**DP**	**ρ**	**p-valor**
Idade	69,38	7,73	-0,125	0,496
Tempo de estudo (em anos)	10,41	5,6	-0,103	0,575
MEEM	26,22	2,49	-0,249	0,17
Repetição de palavras^*^	-2,52	3,15	-0,072	0,694
Fluência verbal semântica*	-0,41	0,79	-0,262	0,148
Fluência verbal fonológica*	-0,84	1,23	-0,137	0,453
Compreensão do discurso oral*	0,11	0,87	-0,037	0,842

* z-score conforme escolaridade

Legenda: M = média; DP = desvio padrão; EAT-10 = Eating Assessment Tool; MEEM = Mini Exame do Estado Mental

Nas tarefas de linguagem realizadas por meio das tarefas da Bateria MTL-Brasil, como repetição de palavras, fluência verbal fonológica e semântica e compreensão oral do discurso, apenas a última tarefa citada apresentou valores dentro da normalidade para todos os participantes, conforme a escolaridade, sendo todas as demais alteradas para 26 participantes.

Ademais, dos 13 participantes que apresentaram queixa alimentar, apenas um não apresentou alterações nos testes de linguagem realizados – todos os outros, 12, apresentaram z escore indicativo de alteração em ao menos uma tarefa de linguagem aplicada.

Conforme observado anteriormente, constatou-se nos resultados do EAT-10 que quatro participantes apresentaram alterações em relação à normalidade. Ao comparar as questões relacionadas à alimentação com as de cognição e linguagem, todos esses participantes com EAT-10 alterado também exibiram escores alterados no MEEM, além de pelo menos uma tarefa de linguagem com um z escore indicativo de alteração.

No entanto, ao se correlacionar os dados obtidos no EAT-10 com a idade, escolaridade em anos, resultado do teste de rastreio cognitivo e tarefas de linguagem, não foi possível verificar significância estatística.

No teste não paramétrico de Mann-Whitney, conforme demonstrado na Tabela 4, ao selecionar os idosos que compõem o grupo com alterações nos resultados obtidos no EAT-10 e os que não possuem, não foi possível observar diferença estatisticamente significante em relação as variáveis de idade, escolaridade, resultado obtido no MEEM e nas tarefas da Bateria MTL-Brasil como repetição de palavras, fluência verbal semântica, fluência verbal fonológica e compreensão do discurso oral, entre um grupo e outro, conforme p-valores demonstrados.

**Tabela 4:** Teste de Mann-Whitney para grupo com EAT-10 alterado e queixa de necessidade de comida modificada com as demais variáveis de estudo (n = 32)

	Necessidade de comida modificada				EAT-10			
	**M**	**DP**	**U**	**p-valor**	**M**	**DP**	**U**	**p-valor**
Idade	68,5	8,7	99,0	0,346	65,5	3,5	38,5	0,317
Tempo de estudo (em anos)	9,1	5,0	96,5	0,296	12,5	3,7	41,0	0,389
MEEM	25,4	2,4	75,0	0,060	26,5	1,7	53,0	0,863
Repetição de palavras^*^	-2,3	3,1	118,0	0,832	-1,2	1,5	43,5	0,474
Fluência verbal semântica*	-0,6	0,7	92,5	0,234	-0,4	0,6	55,0	0,954
Fluência verbal fonológica*	-1,2	1,0	91,0	0,212	-0,8	1,1	56,0	1,000
Compreensão do discurso oral*	0,0	0,8	95,5	0,278	0,3	0,5	49,0	0,687

* z-score conforme escolaridade

Legenda: M = média; DP = desvio padrão; U = valor da estatística de comparação por meio do teste Mann Whitney; EAT-10 = Eating Assessment Tool; MEEM = Mini Exame do Estado Mental

Ademais, também não houve diferença estatisticamente significante ao comparar a queixa de necessidade de comida modificada com os achados de idade, escolaridade, MEEM e tarefas de repetição de palavras, fluência verbal semântica, fluência verbal fonológica e compreensão do discurso oral, onde se verificou ausência de significância estatística, conforme p-valores demonstrados.

Nesse caso, não é possível inferir que a presença de queixa de deglutição possui correlação com a idade e nos resultados obtidos nos testes acima citados, naqueles que possuem essa queixa com os que não a possuem.

Foram elaborados modelos de regressão logística binária para prever variáveis dependentes como sinais de disfagia para líquido, para sólidos, necessidade de comida modificada, dificuldade para ingerir remédios, dificuldade de mastigação e resultado EAT-10 (considerando ponte de corte de alteração), e YSP, a partir das variáveis independentes como idade, gênero, escolaridade, MEEM e tarefas da Bateria MTL-Brasil. Também foi elaborado modelo de regressão linear múltipla para prever a variável dependente EAT-10 a partir das variáveis independentes como idade, gênero, escolaridade, MEEM e Bateria MTL-Brasil. Nenhum modelo obteve resultados estatisticamente significantes.

## DISCUSSÃO

Uma vez que o objetivo do estudo foi descrever os achados de deglutição e as variáveis sociodemográficas, desempenho cognitivo e de linguagem, bem como verificar a associação entre estes fatores, observou-se que quase metade da amostra dos participantes apresentou queixa alimentar, bem como foi observado que 68% indivíduos apresentaram MEEM alterado e 81% apresentaram alterações em alguma(s) tarefa(s) de linguagem. Apesar da ausência de associações estatisticamente significantes que comprovem a correlação destas variáveis, – resultados estes que serão discutidos a seguir – foi possível observar que os mesmos idosos que apresentavam queixas de dificuldades na alimentação, também apresentavam escores sugestivos de alteração cognitiva e linguística.

Quanto à alimentação, os 32 participantes relataram média de apenas quatro refeições ao dia, compatível com um estudo realizado no Sul do Brasil com a mesma média encontrada, que considera essa quantidade reduzida para atender as necessidades nutricionais do ser humano^(21)^. Já sobre as queixas alimentares, quase metade da amostra (40%) referiu necessidade de comida modificada ao se alimentar, bem como queixas de dificuldade para engolir sólidos, líquidos, medicamentos e de mastigar. Alguns fatores descritos previamente podem estar relacionados à alimentação como a diminuição de apetite com o avançar da idade, diminuição de olfato e do paladar, efeito de medicações no paladar e no apetite, dificuldades relacionadas ao preparo das refeições por outros ou até mesmo ao desânimo em preparar suas próprias refeições por residirem sozinhos, além de fatores sociodemográficos como dinheiro para comprar alimentos^(21,22)^.

Além disso, essas dificuldades alimentares relatadas no presente estudo podem ser justificadas pela idade, variável demográfica que é fator de risco para o desenvolvimento de ajustes alimentares^(4)^. Estudos mostram que cerca de 13% das pessoas acima de 65 anos apresentam dificuldades de deglutição e esse número pode subir para 50% ao considerar idosos com comprometimentos cognitivos^(22)^. Essa dificuldade de deglutição, caracterizada como presbifagia, acarreta alterações no processo que não geram, obrigatoriamente, penetração/aspiração laringotraqueal, apesar de aumentarem o seu risco de ocorrência. No entanto, essas dificuldades podem impactar em aspectos nutricionais^(23)^.

Entre as queixas relatadas pelos idosos do presente estudo, houve maior frequência, 40%, de necessidade de comida modificada com ajuste de consistência alimentar para sólidos macios e úmidos. Esta necessidade pode estar relacionada com fatores como força de língua diminuída, aumento do tempo de mastigação, diminuição da pressão orofaríngea para ejeção do bolo alimentar, maior presença de resíduos em cavidade oral e em faríngea, maior aparecimento de penetração laríngea de clareamento espontâneo e diminuição na motilidade esofágica podem ser observados em idosos^(24)^.

Além disso, um estudo recente que avaliou a deglutição de indivíduos idosos por meio do exame de videofluoroscopia, observou que quase metade dos participantes com mais de 85 anos possuíam disfagia, mesmo que sem doenças de base prévia, e que a presbifagia se justificava pela sarcopenia relacionada à idade avançada^(25)^. Foi possível observar ainda, que, quanto mais idosa, mais alterações de deglutição a pessoa apresentava, com um efeito “acumulativo” destas alterações^(25)^. Apenas quatro idosos do presente estudo apresentaram autoavaliação no EAT-10 com escore alterado para presença do risco de disfagia. A menor proporção de idosos com sinais sugestivos de disfagia neste estudo pode ser justificada pela idade dos participantes da amostra, aspecto que será discutido adiante.

Destaca-se, ainda, que apesar do presente estudo ter apresentado poucos participantes com alterações no EAT-10, um estudo realizado com a aplicação deste protocolo com idosos típicos, encontrou correlação positiva entre a idade e a pontuação obtida no EAT-10, demonstrando a sua relevância para a identificação de dificuldades de deglutição^(26)^. Mais uma vez, nos resultados do nosso estudo, não houve correlação estatisticamente significante entre os escores do EAT-10 e a idade. Essa diferença em relação à literatura pode estar relacionada às características etárias de nossa amostra, composta predominantemente por idosos mais jovens e com apenas três idosos com mais de 85 anos. Assim, os resultados devem ser considerados predominantemente para idosos com idade até 85 anos, diante da limitação da composição da amostra com idosos mais velhos e também ao número amostral pequeno.

Ademais, a menor proporção de idosos com risco de disfagia pode estar relacionada à média de escolaridade dos participantes^(27)^, que foi de dez anos; também à renda que se apresenta acima da média da população^(28)^ e à amostra composta em sua maioria por mulheres, variáveis associadas a melhores condições de saúde^(9,10)^.

Acrescenta-se, ainda, que quase todos os indivíduos com queixas alimentares apresentaram escore no MEEM sugestivo de alteração segundo os dados normativos brasileiros^(18)^, bem como alteração em alguma tarefa de linguagem da Bateria MTL. Já se sabe que a cognição faz parte do processo de controle da deglutição no processo de engolir, quando se trata principalmente da fase oral da deglutição, bem como no reconhecimento de alimentos e das sensações como prazer alimentar e à saciedade e que indivíduos com alterações cognitivas diagnosticadas como no caso de demências podem apresentar prejuízos na segurança alimentar e nutrição^(27)^. Ainda em idosos típicos, as alterações de deglutição esperadas se referem mais ao envelhecimento muscular em si do que ao controle cognitivo^(24)^. O estudo não identificou correlação estatisticamente significante entre o quadro de alimentação e deglutição e o quadro cognitivo e de linguagem. Assim, acredita-se que as queixas alimentares e os escores do EAT-10 sugestivos de alteração da deglutição obtidos no presente estudo estejam relacionados principalmente ao quadro muscular, uma vez que os idosos que apresentaram escore no MEEM sugestivo de declínio cognitivo apresentaram pontuações muito próximas do dado normativo.

Estudos recentes sobre presbifagia têm direcionado a atenção para as mudanças anatomofisiológicas associadas ao envelhecimento, todavia, há um questionamento crescente acerca do foco exclusivo nessas transformações sem considerar devidamente a influência das habilidades cognitivas nesse processo^(24-27)^. Embora as alterações estruturais e funcionais do sistema digestivo, desde a boca até o estômago, desempenhem um papel significativo na presbifagia, negligenciar a interação entre os aspectos cognitivos e a deglutição pode limitar a compreensão abrangente dos desafios enfrentados pelos idosos.

No entanto, apesar do conhecimento descrito na literatura ao relacionar o processo do envelhecimento muscular em si com os achados de deglutição e, apesar da presente pesquisa não apresentar dados estatisticamente significantes ao correlacionar as variáveis, estudos demonstram a presença da correlação entre a cognição e a deglutição. Um estudo realizado por *Maniaci e colaboradores* ^(29)^, evidenciou que no grupo com MEEM alterado, 44% apresentavam disfagia, com dados estatisticamente significativos quando comparados ao grupo controle; além disso, os sujeitos do grupo controle que obtiveram melhores pontuações no MEEM (mesmo que já dentro da normalidade esperada para a escolaridade e idade) apresentaram melhor desempenho de deglutição^(29)^.

Especificamente a respeito da cognição, quanto aos resultados do MEEM, 22 participantes apresentaram escores abaixo do esperado para a escolaridade^(18)^. Outros estudos realizados com idosos neurotípicos também encontraram escores sugestivos de alteração cognitiva ao aplicar o MEEM^(30,31)^. A presente pesquisa destaca 68% de idosos com escore sugestivo de alteração cognitiva, enquanto os outros estudos citados anteriormente identificaram, respectivamente, 55% e 16% de idosos com este mesmo quadro clínico. Por outro lado, um estudo desenvolvido com idosos da mesma região estudada também identificou alta prevalência de idosos, 80%, com quadro clínico sugestivo de alteração cognitiva, também por meio da aplicação do MEEM^(32)^. Assim, acredita-se que as características sociodemográficas podem justificar o resultado cognitivo, mas também é necessário referir que os dados normativos do instrumento foram obtidos com amostra de outra região do país, o que pode destacar ainda mais a relevância das variáveis demográficas na análise do quadro clínico cognitivo. Além disso, a aplicação dos testes de maneira *online* pode gerar dificuldades de compreensão suficientes para a obtenção do resultado alterado, limitação do presente estudo.

É necessário destacar ainda, que estudos mostram que obter resultados sugestivos de alteração no MEEM não significa necessariamente que esses idosos evoluirão com demência, uma vez que o desenvolvimento dessas alterações depende também das interferências nas atividades de vida diária, convivência familiar e humor^(30)^.

Ademais, a presente amostra foi composta por idosos com média de escolaridade de 10,00±5,60 anos e renda média melhor em relação aos brasileiros no geral, fator esse que poderia justificar um melhor desempenho linguístico-cognitivo, baseando-se na teoria da reserva cognitiva, e dessa forma, interferir de maneira positiva no desempenho de deglutição, sendo até mesmo fator de compensação de possíveis dificuldades alimentares, uma vez que a reserva cognitiva traz consigo um atraso nos sintomas do declínio cognitivo relacionado à doença^(33)^.

É importante destacar que, uma vez que a presente pesquisa se realizou de maneira *online*, esse fator pode ter influenciado nas respostas obtidas, uma vez que os protocolos escolhidos foram inicialmente descritos para aplicação presencial, além de que, o ambiente virtual no contexto do idoso de média e baixa escolaridade também pode ser fator dificultador de desempenho na realização dos testes devido pouca intimidade com a tecnologia, mesmo que essa variável tenha sido controlada a partir da possibilidade da presença de um acompanhante e mesmo que os resultados obtidos pelos participantes tenham sido compatíveis com a literatura previamente encontrada.

Além disso, uma vez que todos os protocolos realizados se trata de testes de rastreio, dados mais robustos e detalhados sobre a cognição e seus aspectos específicos (como funções executivas, memória de trabalho, tipos de atenção, dentre outros), bem como exames objetivos da deglutição, devem ser levados em consideração, uma vez que alteração em alguma destas habilidades, poderiam interferir no processamento cognitivo da deglutição de maneira já sutil, conforme demonstra a literatura^(29)^.

Sugere-se ainda, que estudos futuros, principalmente na modalidade virtual, sejam realizados com um número maior de participantes.

## CONCLUSÃO

O presente estudo, ao correlacionar os achados de deglutição, linguagem e cognição, não obteve resultados estatisticamente significantes. Apesar disso, observou-se que os idosos com queixa de deglutição também apresentaram resultados sugestivos de alteração cognitiva e de linguagem nos testes realizados.
